# Germline Testing in Breast Cancer: A Single-Center Analysis Comparing Strengths and Challenges of Different Approaches

**DOI:** 10.3390/cancers17091419

**Published:** 2025-04-24

**Authors:** Monica Marabelli, Mariarosaria Calvello, Elena Marino, Chiara Morocutti, Sara Gandini, Matteo Dal Molin, Cristina Zanzottera, Sara Mannucci, Francesca Fava, Irene Feroce, Matteo Lazzeroni, Aliana Guerrieri-Gonzaga, Francesco Bertolini, Bernardo Bonanni

**Affiliations:** 1 Division of Cancer Prevention and Genetics, IEO, European Institute of Oncology, IRCCS, 20141 Milan, Italy; 2 Laboratory of Medical Genetics, Cytogenetics and Molecular Genetics, IEO, European Institute of Oncology, IRCCS, 20141 Milan, Italy; 3 Department of Experimental Oncology, IEO, European Institute of Oncology, IRCCS, 20141 Milan, Italy; 4 Laboratory of Hematology-Oncology, IEO, European Institute of Oncology, IRCCS, 20141 Milan, Italy

**Keywords:** germline genetic testing, breast cancer, single gene testing, multigene panel testing, primary findings, secondary findings

## Abstract

Background/Objectives: Compared to single gene testing (SGT), multigene panel testing (MGPT) improves pathogenic variants (PVs) detection. However, MGPT yields complex results, including secondary findings, heterozygous PVs in recessive genes, low-penetrance PVs, and variants of uncertain significance. We reported our mono-institutional experience of germline testing in breast cancer (BC), comparing SGT and MGPT. Methods: We retrospectively analyzed clinical and molecular data from 1084 BC patients: 308 underwent SGT (*BRCA1*/*BRCA2*) and 776 MGPT (for 28 cancer-related genes). We compared these approaches regarding the genetic classification of the findings (positive, uncertain, uninformative) and their impact on clinical management (primary findings (PFs); complex and inconclusive results). Additionally, we described clinical features supporting one approach over the other and focused on copy number variation (CNV) frequency in non-*BRCA* genes. Results: We found ≥1 PV in 165 patients (165/1084 = 15.2%), including 91 in *BRCA1*/*BRCA2* (91/1084 = 8.4%), with 42 identified by SGT (42/308 = 13.6%) and 49 by MGPT (49/776 = 6.3%). MGPT detected PVs in non-*BRCA* genes in 74 patients (74/776 = 9.5%), including 40 PFs. Overall, MGPT identified 89 PFs (89/776 = 11.5%). We observed complex results in 21 patients (21/308 = 6.8%) with SGT and in 300 (300/776 = 38.7%) with MGPT. Compared to MGPT, SGT detected a similar percentage of PFs (13.6% vs. 11.5%) but a significantly reduced percentage of complex results (6.8% vs. 38.7%) (*p* < 0.001). Triple-negative BCs prevailed in *BRCA1* carriers, while ER-positive BCs were more prevalent in *ATM*/*CHEK2* carriers. Concerning non-*BRCA* genes, MGPT detected CNVs in *PALB2*, representing 20% of PVs in this gene. Conclusions: Although MGPT increases hereditary BC detection, its complexity requires clear guidelines for optimal clinical management and strategies for merging the benefits of SGT and MGPT.

## Simple Summary

The debate over choosing single gene testing (SGT) or multigene panel testing (MGPT) in cancer genetics, particularly for breast cancer (BC), has recently shifted in favor of MGPT. This shift reflects the complex architecture of genetic BC risk, involving high/moderate penetrance genes. However, MGPT still presents challenges, requiring guidelines or strategies to leverage the benefits of both approaches. In our monocentric retrospective study of 1084 BC patients, we found that MGPT offers advantages over SGT, but it also raises questions about managing complex results. We explored patient features supporting MGPT. Furthermore, we investigated the frequency of copy number variations in non-*BRCA* genes, previously underestimated due to the limitations of next generation sequencing and now addressed through advanced bioinformatics protocols. Our findings regarding a large cohort of patients endorse MGPT as a valuable approach for germline testing in BC, nevertheless highlighting the need for further refinement to optimize its clinical applications.

## 1. Introduction

Germline genetic testing in breast cancer (BC) has evolved significantly in recent years, enhancing the identification of hereditary cases [1]. Traditionally, *BRCA1* and *BRCA2* (*BRCA*) have been considered the primary genes responsible for hereditary BC [2], with *PALB2* now also recognized as a high-penetrance BC gene [3,4]. However, other BC susceptibility genes have recently been identified, including moderate-penetrance genes (such as *ATM*, *BARD1*, *CHEK2*, *RAD51C*, and *RAD51D*), as well as other genes already known for their role in rare cancer predisposition syndromes (CPS), which also include BC susceptibility (such as *CDH1*, *PTEN*, *STK11*, and *TP53*) [3,4]. Notably, the *CDH1* gene, historically responsible for hereditary diffuse gastric cancer, has recently emerged as associated with lobular BCs, even in the absence of a personal or family history of gastric cancer [5]. It is now well-established that the genetic basis of hereditary BC involves a complex risk architecture, encompassing pathogenic or likely pathogenic variants (PVs) in high- and moderate-penetrance genes and/or combinations of low-penetrance variants [6].

Consequently, driven by technological advancements, germline testing in BCs has increasingly shifted from a single gene testing (SGT) approach (i.e., the analysis of one or a few genes associated with a specific clinical suspicion or patient phenotype) to a multigene panel testing (MGPT) approach (i.e., the simultaneous analysis of multiple genes). MGPT is particularly well-suited for conditions characterized by high genetic heterogeneity, as well as clinical presentations suggestive of different CPSs or those that do not clearly indicate a need for genetic testing [7]. Notably, the MGPT approach offers several advantages, including increased detection of PVs, reduced turnaround time, and reduced costs [8].

However, beyond primary findings (PFs) (i.e., PVs consistent with the indication for genetic testing), MGPT can yield complex results that are challenging to interpret or of limited utility [9,10]. For instance, MGPT may occasionally detect secondary findings (SFs) (i.e., PVs identified in genes unrelated to the patient’s phenotype or to the primary purpose of the testing) [11]. In BC patients, MGPT can identify PVs in genes whose association with BC risk is debated, such as mismatch repair (MMR) genes (i.e., *MLH1*, *MSH2*, *MSH6*, and *PMS2*) responsible for Lynch syndrome (LS). More rarely, other genes such as *CDKN2A*, associated with melanoma and pancreatic carcinoma syndrome, may be identified by MGPT [12,13].

Similar to PFs, SFs represent actionable results, since they are generally identified in genes with an evolving evidence base and/or an evolving standard in medical practice [14]. However, they still constitute a challenge in clinical management, as it remains unclear whether the guidelines developed according to studies on highly selected individuals/families are applicable for carriers of SFs.

Along with SFs, other challenging results of MGPT include variants of uncertain significance (VUSs), PVs in moderate-penetrance genes, and findings of limited clinical relevance. The latter category includes low-penetrance PVs (lowPVs) and heterozygous PVs in recessive genes (hetPVs), for which there is no consensus regarding their association with cancer risk in the heterozygous state (e.g., *MUTYH*, *NTHL1*, *NBN*, *RAD50*, *MRE11*, and others) [10]. LowPVs are generally associated with an odds ratio between 1 and 2, meaning that their contribution to disease risk is modest and often depends on additional genetic and environmental factors. However, the interpretation of lowPVs, sometimes not clearly differentiated from risk alleles, remains problematic due to the absence of clear-cut standardized terminology and established risk thresholds [15].

The MGPT approach also presents inherent limitations, including sensitivity constraints. Previously, copy number variations (CNVs) were often undetected by next generation sequencing (NGS). Current improvements in analytical protocols have considerably mitigated this issue. However, the historical underestimation or inadequate detection of CNVs in non-*BRCA* genes significantly affected diagnostic and research efforts in hereditary BCs, mainly when orthogonal methods like multiplex ligation-dependent probe amplification were not applicable or performed [16].

Regarding potential genotype–phenotype correlations, recent data indicate that *BRCA1*, *PALB2*, *BARD1*, *RAD51C*, and *RAD51D* are more strongly associated with triple-negative tumors (TNBC). In contrast, *CHEK2* and *ATM* genes are linked to luminal type tumors, i.e., estrogen receptor (ER)-positive BCs [4]. Additionally, BCs diagnosed in *BRCA2* carriers are predominantly luminal B, a molecular subtype that is ER-positive, progesterone receptor-negative, and either HER2-positive or HER2-negative, with either high or low Ki-67 expression [17].

In this retrospective study of BC patients who underwent germline genetic testing, we aimed first to compare the outcomes of SGT and MGPT across two key dimensions: the genetic classification of the findings (positive, uncertain, uninformative) and their impact on clinical management. Indeed, comparing SGT and MGPT can provide valuable insights into their respective strengths and limitations and the optimal clinical applications of each approach. Furthermore, we characterized the most distinctive features of high-risk genes, such as *BRCA1* and *BRCA2*, that justify an initial SGT approach and those that might warrant MGPT as the preferred testing strategy. Finally, we focused on the frequency of CNVs detected by MGPT in non-*BRCA* genes within our study population.

## 2. Materials and Methods

### 2.1. Study Population

We retrospectively analyzed clinical and molecular data for 1084 BC patients undergoing genetic testing at the European Institute of Oncology (IEO) for unknown germline variants in cancer-associated genes. Patients were consecutively referred to the Division of Cancer Prevention and Genetics for oncogenetic counseling, and germline testing was carried out at the Laboratory of Medical Genetics, Cytogenetics, and Molecular Genetics between January 2021 and June 2023. Most patients met the selection criteria in place at the time of testing for BC predisposition and/or other rare CPSs.

The following data were collected for all patients: personal and family history, clinical and histopathological features of BC (age at diagnosis, histotype, bilaterality, hormone receptor status, HER2 status, Ki-67 status, and grading), and results of genetic testing. Specifically, TNBC was defined as follows: estrogen receptor < 5%, progesterone receptor < 5%, and absence of HER2 overexpression (immunohistochemistry score = 0 or score = 1+/2+ with negative fluorescence in situ hybridization for *ERBB2* amplification). Clinical and genetic data were stored in a dedicated institutional database. Additional data were retrieved using medical records and pathology reports.

All participants provided their written informed consent before undergoing molecular analysis and consented to the use of their data for research purposes. The study was approved by the IEO Institutional Review Board (code UID 4376).

### 2.2. Molecular Analyses

For each patient included in the study, genomic DNA was isolated from 400 μL of whole peripheral blood using a MagCore Super Automated Nucleic Acid Extractor (Diatech Pharmacogenetics srl, Jesi, Italy) and quantified with a Qubit^®^3.0 fluorometer (Life Technologies, Carlsbad, CA, USA).

Two different NGS panels were utilized:

- In 308 patients, genetic testing was carried out via an SGT approach using the Devyser *BRCA* NGS test (Devyser AB, Stockholm, Sweden), which allows for the analysis of *BRCA* genes.
- In the remaining 776 patients, analysis was performed through an MGPT approach with a custom Hereditary Cancer Solution panel (SOPHiA GENETICS, Geneva, Switzerland), which includes 28 cancer predisposition genes (*ABRAXAS1*, *APC*, *ATM*, *BARD1*, *BRCA1*, *BRCA2*, *BRIP1*, *CDH1*, *CDK4*, *CDKN2A*, *CHEK2*, *EPCAM*, *MLH1*, *MRE11A*, *MSH2*, *MSH6*, *MUTYH*, *NBN*, *PALB2*, *PIK3CA*, *PMS2*, *PTEN*, *RAD50*, *RAD51C*, *RAD51D*, *STK11*, *TP53*, *XRCC2*) and the pseudogene *PMS2CL*. This in-house validated panel initially covered 26 genes, but it was customized with two additional genes for familial melanoma predisposition (*CDKN2A* and *CDK4*).

Library preparation was performed under standard conditions. Samples were sequenced using a MiSeq platform (Illumina Inc., San Diego, CA, USA), following the manufacturers’ instructions.

Depending on the panel used, the CE-IVD bioinformatic tools Amplicon Suite (Devyser AB, Stockholm, Sweden) or SOPHiA DDM (SOPHiA GENETICS, Geneva, Switzerland) were employed for NGS data analysis. The sequencing reads were aligned against the human reference genome (hg19/GRCh37). For both NGS systems, a high mean read quality score (Q score > 30) and a minimum read depth of 50× for each amplicon were established to ensure high-confidence variant calling. Full coverage (100%) of the coding regions was obtained.

The NGS analytical protocols used for both panels demonstrated a sensitivity of >99% and a specificity of >99% in identifying single nucleotide variants (SNVs), small insertions/deletions (indels), and large deletions/duplications (CNVs). The analyses included all the coding exons and intron/exon boundaries (+/−20 bp) of each investigated gene.

All the reported variants underwent confirmation by Sanger sequencing or multiplex ligation-dependent probe amplification (MLPA kits: MRC-Holland, Amsterdam, The Netherlands) from a second independent blood sample to rule out false-positive results.

Genetic variants emerging from the analysis were divided into five classes in accordance with the International Agency for Research on Cancer (IARC) recommendations [18]. Variant classification was assessed according to the American College of Medical Genetics and Genomics and the Association for Molecular Pathology (ACMG/AMP) guidelines [19]. Moreover, gene-specific criteria, developed by ClinGen Variant Curation Expert Panels (https://clinicalgenome.org, accessed on 23 April 2025), were applied, when available. Useful information for variant interpretation was retrieved by using the following databases and tools: the ClinVar database (https://www.ncbi.nlm.nih.gov/clinvar, last accessed on 25 February 2025), the *BRCA* Exchange (https://brcaexchange.org, last accessed on 25 February 2025), the Leiden Open Variation Database (https://www.lovd.nl/3.0/home, last accessed on 25 February 2025), the International Society for Gastrointestinal Hereditary Tumors database (http://insight-database.org, last accessed on 25 February 2025), and VarSome Premium (http://landing.varsome.com, last accessed on 25 February 2025). All variants were named following the nomenclature of the Human Genome Variation Society (HGVS v.21.1, https://hgvs-nomenclature.org, last accessed on 25 February 2025). In this study, both pathogenic (C5) and likely pathogenic (C4) variants are labeled as PVs.

### 2.3. Classification of Genetic Testing Results

**Figure 1:**
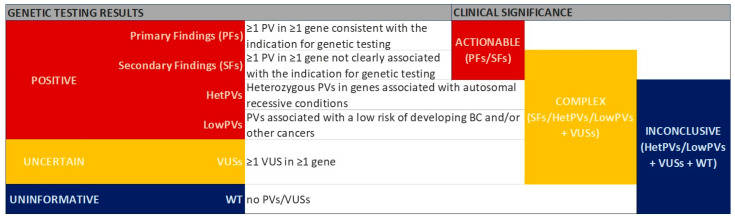
Classification of genetic testing results and their clinical significance. BC, breast cancer; hetPV, heterozygous PV in genes associated with autosomal recessive conditions; lowPV, low-penetrance PV; PF, primary finding; PV, likely pathogenic or pathogenic variant; SF, secondary finding; VUS, variant of uncertain significance; WT, wild-type.

We classified the genetic testing results according to the main findings (positive, uncertain, and uninformative) and their clinical significance (actionable, complex, and inconclusive), as summarized in Figure 1.

To more clearly define positive results based on their clinical significance, we specifically distinguished PFs, SFs, hetPVs, and lowPVs. In addition, we established the following classifications: (a) complex—the detection of SFs, hetPVs, lowPVs, or at least one VUS in the absence of PVs; (b) actionable—the detection of PFs or SFs; and (c) inconclusive—the detection of hetPVs, lowPVs, VUSs, or wild-type (WT) results.

Finally, as reported in Table S1, we classified the genes into the following types:

- *BRCA* genes—*BRCA1* and *BRCA2*;
- Other non-*BRCA* BC susceptibility genes—genes known to be associated with BC (i.e., *ATM*, *BARD1*, *CDH1*, *CHEK2*, *PALB2*, *PTEN*, *RAD51C*, *RAD51D*, *STK11*, *TP53*);
- Genes responsible for other CPSs not strictly known to be associated with BC risk (such as *APC*, *CDK4*, *CDKN2A*, *MLH1*, *MSH2*, *MSH6*, *MUTYH*, *PMS2*, *EPCAM* deletions of the 3′ region);
- Genes included in the panel and not clearly associated with BC risk (*ABRAXAS1*, *BRIP1*, *MRE11A*, *NBN*, *PIK3CA*, *RAD50*, *XRCC2*, point mutations in *EPCAM*).

### 2.4. Statistical Analyses

Clinical features and genetic test results were described by calculating frequencies and percentages for the categorical variables and the medians with interquartile ranges (IQRs) for the continuous variables. Fisher’s exact test or the Chi-squared test were used, as appropriate, to compare the results between the SGT and MGPT approaches, as well as the clinicopathological characteristics between the two cohorts or between carriers and non-carriers of PVs in different genes. To compare age at diagnosis, we utilized Wilcoxon–Mann–Whitney or Kruskal–Wallis tests.

Multivariable logistic models were applied to investigate whether carriers of PVs exhibited a significantly different association with TNBC and ER-positive BC, adjusting for significant potential confounders, such as bilateral tumor, grading, and age. Adjusted odds ratios (ORs) and 95% confidence intervals (CIs) are reported. A *p*-value of <0.05 was considered statistically significant. We also applied the Benjamini–Hochberg procedure in order to calculate the false discovery rate (FDR). We highlighted the results which remained significant after adjustment for multiple testing. All analyses were conducted using R version 4.0.0.

## 3. Results

### 3.1. Genetic Testing Results

Overall, regardless of the genetic testing approach, the analyses yielded the following outcomes: a positive result in 165 patients (165/1084 = 15.2%); an uncertain result in 287 patients (287/1084 = 26.5%); and an uninformative result in the remaining 632 patients (632/1084 = 58.3%).

#### 3.1.1. SGT Results

Table 1 summarizes the results obtained from 308 patients who underwent genetic testing using an SGT approach. The test yielded a positive result in 42 subjects (13.6%). All patients displayed a single PV. More specifically, 32 patients (10.4%) were found to carry a PV in *BRCA1*, and 10 patients (3.2%) exhibited a PV in *BRCA2*. All these PVs have been considered as PFs. In addition, 21 patients (6.8%) were found to have at least one VUS in *BRCA1* or *BRCA2* in the absence of PVs. In the remaining 245 subjects (79.5%), genetic testing was uninformative, as no PVs nor VUSs were identified in the *BRCA* genes.

#### 3.1.2. MGPT Results

Table 1 and Table S2 summarize the results obtained from 776 patients who underwent genetic testing using an MGPT approach. The test gave a positive result in 123 subjects (15.9%). In total, 133 PVs were identified in these patients, since 10 subjects (1.3%) were found to harbor two PVs in the same or different genes.

Overall, at least one PV in a *BRCA* gene was detected in 49 patients (6.3%). Specifically, a single PV in *BRCA1* was found in 13 patients (1.7%) and a single PV in *BRCA2* in 31 patients (4.0%). One patient (0.1%) was found to carry a PV both in *BRCA1* and in *BRCA2*, while another patient (0.1%) carried two different PVs in *BRCA2*. The remaining three patients had a PV in *BRCA2* and a second PV in one of the following genes: *CHEK2* (0.1%), *RAD51C* (0.1%), or *APC* (0.1%).

PVs in non-*BRCA* BC susceptibility genes were identified in 41 patients (5.3%) without PVs in *BRCA* genes. Of these, 40 patients carried a single PV: 10 cases (1.3%) in *PALB2*, 9 cases (1.2%) in *ATM*, 14 cases (1.8%) in *CHEK2*, 3 cases (0.4%) in *CDH1*, 3 (0.4%) in *RAD51C*, and 1 (0.1%) in *BARD1*. Additionally, one patient (0.1%) was found to harbor PVs in both *ATM* and *RAD51D*.

PVs in other CPS genes were identified in 33 patients (4.3%) who did not carry PVs in *BRCA* genes. Among these, 29 patients carried a single PV: 2 cases (0.3%) in *BRIP1*, 1 (0.1%) in *CDKN2A*, 4 (0.5%) in *RAD50*, 2 (0.3%) in *MLH1*, 1 (0.1%) in *PMS2*, 6 (0.8%) in *APC*, and 13 (1.7%) in *MUTYH*. In contrast, four patients harbored two PVs: one (0.1%) in *NBN* and *MRE11A*, one (0.1%) in *CDKN2A* and *MUTYH*, one (0.1%) in *APC* and *EPCAM*, and one (0.1%) who was compound heterozygous for two PVs in *MUTYH*. Notably, no PVs were identified in nine genes included in the panel, e.g., *TP53*, *PTEN*, or *STK11*.

An uncertain result was obtained in 266 patients (34.3%). A total of 426 VUSs were identified in 313 patients, including 47 carriers of at least one PV, in addition to the identified VUS(s).

Finally, for 387 patients (49.9%), genetic testing was uninformative, as neither PVs nor VUSs were detected in the investigated genes.

#### 3.1.3. Clinical Interpretation of Genetic Testing Results

In the overall study population, we identified a PF in 131 patients (131/1084 = 12.1%). In particular, we detected at least one PV of a *BRCA* gene in 91 cases (91/1084 = 8.4%): 42 identified by SGT (42/308 = 13.6%) vs. 49 by MGPT (49/776 = 6.3%) (*p* < 0.001). In addition, MGPT found PFs for other 40 patients, including 35 carriers of 36 PVs in non-*BRCA* BC susceptibility genes, such as *ATM*, *BARD1*, *CDH1*, *CHEK2*, *PALB2*, *RAD51C*, and *RAD51D*. Notably, we identified a PV in *CDH1* gene in a patient with an early-onset lobular BC and a positive family history of breast and gastric cancers. By definition, such PVs in non-*BRCA* BC susceptibility genes are also classified as PFs. Moreover, MGPT identified seven PVs in CPS genes in five patients with a consistent clinical picture. Accordingly, these five results were again classified as PFs. Specifically, three BC patients with a personal/family history of LS-related cancers were found to be carriers of two PVs in *MLH1* and one PV in *PMS2*, respectively. Notably, all three patients developed colorectal cancers, with immunohistochemical patterns of MMR proteins consistent with the genetic results. In another patient, MGPT detected two PVs in *MUTYH*, confirmed to be in trans through segregation analysis performed in relatives. This BC patient presented a typical picture of familial adenomatous polyposis type 2 associated with *MUTYH*, with both attenuated adenomatous polyposis and colorectal cancer. Finally, a PV in *CDKN2A* emerged from MGPT in a BC patient with a positive family history of melanoma who also carried an additional heterozygous PV in *MUTYH*.

By MGPT, we detected PVs classifiable as SFs in five patients (0.6%). Two of them were carriers of a PV in the *CDH1* gene. Although both showed a positive family history of BC, they did not fully meet the eligibility criteria for *CDH1* genetic testing [5]. One patient proved to be carrier of a PV in *CDKN2A,* even without a personal or family history of melanoma. Finally, two patients harbored a PV in *BRIP1*, although neither had a personal or family history of ovarian cancer (OC).

In 10 patients (1.3%) we identified only lowPVs. The lowPV c.3920T>A (p.Ile1307Lys) of *APC* was detected in eight patients, occurring as a single variant in six cases or with a PV in another gene in two cases. In addition, three different low penetrance missense variants in *CHEK2* were identified in four patients.

A total of 21 hetPVs were detected in 20 patients (2.6%); 14 patients harbored a hetPV in *MUTYH*, including one who also carried a PV in *CDKN2A*. Four patients were found to be carriers of a hetPV in *RAD50*. Finally, one patient carried a nonsense hetPV of *EPCAM* and the lowPV of *APC*, while another one carried hetPVs in both *NBN* and *MRE11A*.

Regarding the MGPT results, we obtained PFs in 89 (11.5%) cases and other types of positive results in 34 (4.4%) cases.

### 3.2. Comparison Between SGT and MGPT Approaches

Table 2 shows the comparison between the SGT and MGPT results based on the genetic classification of the findings (positive, uncertain, uninformative) and their clinical management (PFs, complex, uninformative). In particular, MGPT detected complex results in 300 patients, including 266 cases with at least one VUS identified in the absence of PVs, 5 cases with SFs, 10 cases with only lowPVs, 18 cases with only one hetPV, and one case with both lowPV and hetPV.

For both approaches, we observed a statistically significant difference between the two groups (*p* < 0.001). Compared to MGPT, SGT did not substantially reduce the rate of positive results and PFs, but resulted in a marked reduction in uncertain and complex findings.

### 3.3. Clinical Characterization of BC Patients Based on Genetic Testing Results

Table S3 shows the clinical characteristics of all 1084 BC patients, including a comparison between the SGT and MGPT cohorts. Notably, the two groups proved to be different for specific variables. In particular, a statistically significant difference between the SGT and MGPT cohorts was observed concerning gender (males: 3.6% vs. 0.6%); histotype (no special type: 84.4% vs. 71.0%, lobular: 5.2% vs. 14.3%); grading (G1-G2: 50% vs. 56.6%, G3: 42.2% vs. 31.1%); molecular subtype (luminal: 62.7% vs. 77.4%, TNBC: 28.6% vs. 11.2%); ER status (positive: 64.6% vs. 80.0%); family history of BC (positive: 58.8% vs. 66.1%); and family history of OC (positive: 14.6% vs. 7.3%).

Concerning the SGT cohort, we analyzed the clinicopathological features of BC patients according to the observed results, comparing actionable (i.e., PVs in *BRCA* genes) and inconclusive results (Table S4). High grade (G3) was more frequent in *BRCA* PV carriers than in non-carriers (73.8% vs. 37.2%, *p* < 0.001). Carriers and non-carriers of PVs in *BRCA* genes developed TNBC in 71.4% and 21.8% of cases, respectively (*p* < 0.001). ER-positive BCs were observed in 28.6% of PV carriers vs. 70.3% of non-carriers (*p* < 0.001), while HER2-positive BCs were noted in 2.4% of PV carriers vs. 13.9% of non-carriers (*p* = 0.049). Positive family history of OC was observed in 31.0% of PV carriers and only in 12.0% of non-carriers (*p* = 0.003). Family members with both BC and OC were reported in 7.1% and 1.1% of PV carriers and non-carriers, respectively (*p* = 0.035). However, after adjusting for multiple testing, the differences regarding HER2-positive BCs and the presence of family members with both BC and OC did not remain statistically significant.

To investigate the impact of non-*BRCA* genes on BC clinicopathological features of patients undergoing MGPT, we compared the 45 patients with an actionable result in non-*BRCA* genes (i.e., PFs or SFs) to the 682 patients with an inconclusive outcome (i.e., VUSs, hetPVs, lowPVs, or not carrying any variant in the genes included in the panel) (Table S5). High grade (G3) was more frequent in patients with actionable results (46.7%) than in patients with inconclusive outcomes (28.6%) (*p* = 0.011). TNBC was diagnosed in 20% of patients with actionable results and in 10% of patients with inconclusive outcomes (*p* = 0.039). ER-positive BCs were observed in 66.7% of patients with actionable results and 81.5% of patients with inconclusive outcomes (*p* = 0.036). However, after adjusting for multiple comparisons, only BC grading remained statistically significant.

Multivariable logistic regression models adjusted for significant clinicopathological characteristics (Table 3) confirmed that PVs in *BRCA* genes are significantly associated with the TNBC subtype (OR = 5.97, 95% CI: 2.458–15.818; *p* <0.001) and inversely associated with the ER positive status (OR = 0.29, 95% CI: 0.115–0.677; *p* = 0.006). Similarly, the identification of a PF or SF in non-*BRCA* genes seemed to be associated with TNBC diagnosis (OR = 2.46, 95% CI: 1.055–5.277; *p* = 0.026) and inversely associated with the ER positive status (OR = 0.42, 95% CI: 0.211–0.882; *p* = 0.016). However, after adjusting for multiple testing, associations with TNBC and ER positive remained statistically significant only for the SGT actionable results in *BRCA* genes.

As shown in Table 4, we compared the ER and HER2 receptor status of BCs among patients with PVs in (a) *BRCA* genes, (b) *ATM* or *CHEK2*, (c) *BARD1*, *PALB2*, or *RAD51C*, and (d) those without genetic variants. A statistically significant difference was observed regarding the ER status (*p* = 0.004). Indeed, most patients carrying PVs in *ATM* or *CHEK2* (84.2%) developed ER-positive BCs, while half of the patients carrying PVs in *BARD1*, *PALB2*, or *RAD51C* developed ER-negative tumors. In contrast, with regard to HER2 status, no statistically significant difference was observed among the four groups (*p* = 0.599).

Finally, we compared BC clinicopathological features between *BRCA1* and *BRCA2* carriers. High-grade (G3) tumors were more common in *BRCA1* carriers (73.3%) than in *BRCA2* carriers (51.2%) (*p* = 0.015). TNBC was more frequent in women with PVs in *BRCA1* than in *BRCA2* (77.8% vs. 11.6%, *p* < 0.001). In contrast, the predominant molecular subtype in *BRCA2* carriers was luminal B HER2-negative (Figure S1). Most *BRCA2* carriers (86.0%) developed ER-positive BC, compared to 20.0% of *BRCA1* carriers (*p* < 0.001). No HER2-enriched BCs (i.e., hormone receptor-negative and HER2-positive tumors) were found in either group.

### 3.4. Overview of the Variants Identified in the Study

A total of 175 PVs were identified in 165 patients, corresponding to 139 unique PVs in 19 genes (Table S6).

Concerning the *BRCA* genes, we found 41 unique PVs in *BRCA1* (i.e., 38 SNVs/indels and 3 CNVs) and 38 unique PVs in *BRCA2* (i.e., 37 SNVs/indels and 1 CNV). The most frequent PV in *BRCA* genes was the nonsense c.7180A>T (p.Arg2394Ter) variant of *BRCA2*. All *BRCA* CNVs consisted of large deletions involving critical domains for protein function and were classified as pathogenic (C5). The most frequent deletion encompassed the 5′UTR and exons 1 and 2 of the *BRCA1* gene. Remarkably, CNVs of *BRCA* genes accounted for 6.5% (6/93) of all *BRCA* PVs.

**Figure 2:**
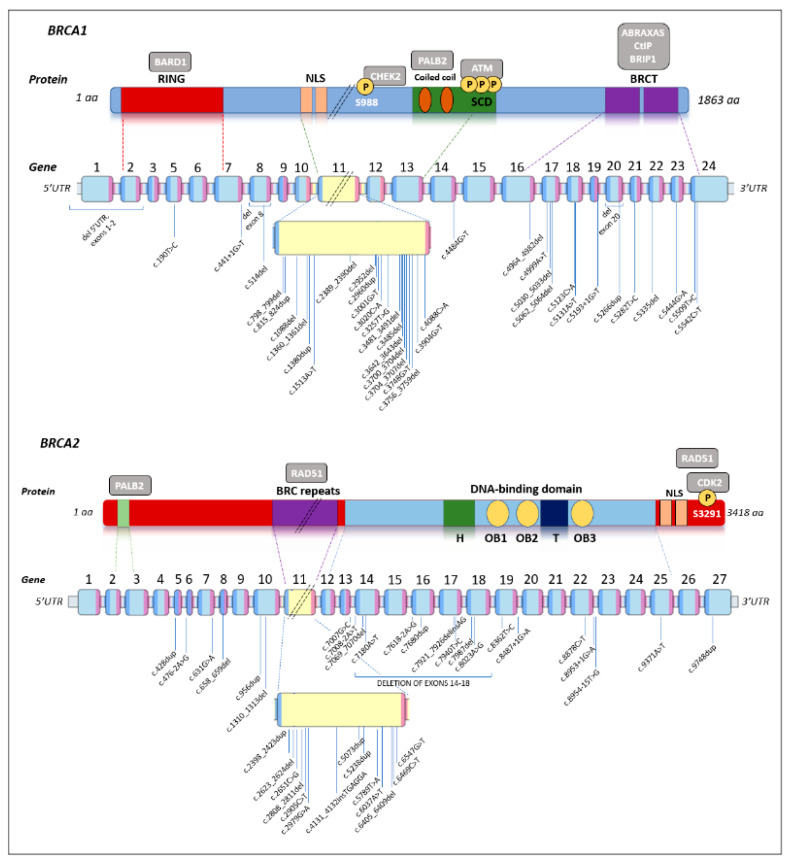
Distribution of all PVs identified in *BRCA1* and *BRCA2* genes. The gene representation has been adapted from *BRCA* Exchange (https://brcaexchange.org/, last accessed on 25 February 2025). In *BRCA1*, exon numbering is shifted by one after exon 3 due to a historical misannotation of an additional exon 4. For both genes, exon 11 does not scale, as it represents >50% of the total sequence.

As shown in Figure 2, germline PVs identified in *BRCA1* and *BRCA2* are evenly distributed throughout the gene, and there were no mutational hotspots.

Regarding the other non-*BRCA* genes, 60 unique PVs were observed across 17 genes (Table S6), including 58 SNVs/indels and two CNVs in *PALB2*, consisting of large duplications of exon 11 and 13, respectively. These duplications were classified as likely pathogenic (C4). Notably, CNVs of *PALB2* accounted for 20% (2/10) of all *PALB2* PVs identified in this study.

The two most frequent PVs identified in the study population were the c.3920T>A (p.Ile1307Lys) of the *APC* gene and c.1187G>A (p.Gly396Asp) of the *MUTYH* gene, each found in eight different patients (8/776 = 1.0%). Another recurrent PV in our population was found to be the c.1100del (p.Thr367MetfsTer15) variant of the *CHEK2* gene, identified in four patients (4/776 = 0.5%).

Regarding variants classified as uncertain, SGT and MGPT detected 27 and 354 unique VUSs, respectively, for a total of 381 unique VUSs in 338 patients.

In particular, 73 unique VUSs (i.e., 27 by SGT and 46 by MGPT) were identified in *BRCA* genes, including 24 in *BRCA1* and 49 in *BRCA2*. Overall, we observed approximately one-third of the *BRCA* VUSs in *BRCA1* (24/73= 32.9%) and more than two-thirds in *BRCA2* (49/73= 67.1%).

**Figure 3:**
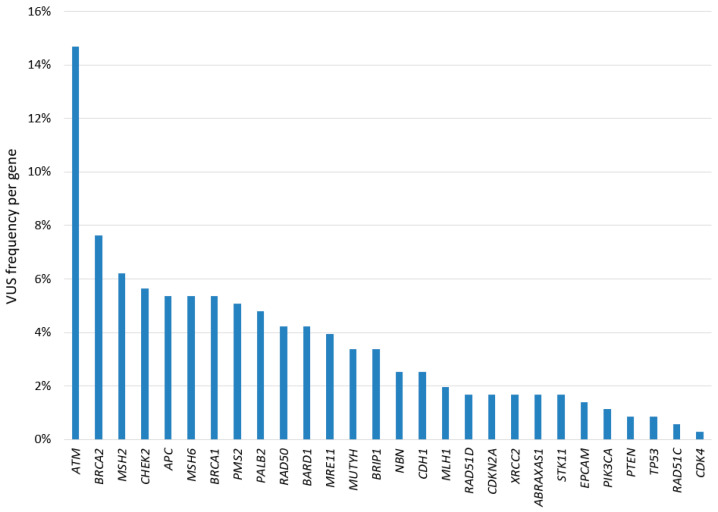
Distribution of unique VUSs identified by MGPT.

In addition to *BRCA* VUSs, MGPT identified 308 unique VUSs in other genes, distributed as shown in Figure 3. The gene with the highest number of VUSs was *ATM* (52/354 = 14.7%), followed by *BRCA2* (27/354 = 7.6%), *MSH2* (22/354 = 6.2%), and *CHEK2* (20/354 = 5.6%).

## 4. Discussion

In this retrospective mono-institutional study of breast cancer patients, we compared germline genetic testing results obtained through SGT and MGPT in terms of the genetic classification of the findings and their impact on clinical management. As expected, the SGT approach yielded a comparable rate of positive results (13.6% vs. 15.9%) and PFs (13.6% vs. 11.5%) when compared to the results for MGPT. However, SGT showed a significant reduction in the percentage of uncertain results (34.3% to 6.8%) and complex results (38.7% to 6.8%).

To date, several authors have emphasized the advantages of MGPT for increasing the detection of PVs [7,20,21]. However, to our knowledge, only a limited number of studies have objectively analyzed which of the identified PVs are clinically actionable and align with the core objectives of genetic testing [22,23]. Our findings confirm that MGPT more commonly yields positive results. However, in over 4% of cases, MGPT did not identify PFs, but rather PVs in genes not strictly related to the phenotype (SFs), or hetPVs or lowPVs, leading to results which are complex to manage or of limited clinical utility. In addition, the higher rate of VUSs observed with MGPT contributed to a significant increase in challenging results, compared to the results for SGT.

In the context of hereditary BCs, PFs identified through germline genetic testing are undoubtedly PVs in genes associated with BC risk, with *BRCA1* and *BRCA2* being the most significant. In this study, *BRCA* genes were analyzed in all BC patients—either through SGT or MGPT—and a PV was identified in 8.4% of cases. This prevalence is higher than that previously reported in unselected BC populations [24,25], likely reflecting the fact that most patients in our cohort were referred for genetic testing based on a personal or family history suggestive of cancer predisposition.

Of note, the frequency of *BRCA* PVs in our study population was primarily driven by patients who underwent SGT (13.6%) compared to MGPT (6.3%). However, it is important to emphasize that the SGT and MGPT cohorts differed significantly in key clinical characteristics. Patients with features typically associated with *BRCA* PVs, such as BC of no special type, TNBC, and family history of OC, were more frequently referred for SGT. In contrast, patients with lobular and/or luminal BC were more commonly directed toward MGPT. Despite these differences, the overall rate of PFs was similar between the two approaches. Indeed, MGPT expanded the spectrum of actionable findings, i.e., in addition to *BRCA* PVs, it identified PVs in non-*BRCA* BC susceptibility genes in approximately 5% of cases, and PVs in CPS genes, either related or unrelated to the patient’s phenotype, in about 1% of patients. These findings suggest that when clinical features strongly indicate BRCA involvement, an SGT approach may be appropriate. Conversely, for features less suggestive of BRCA PVs or more consistent with other CPSs, MGPT should be considered as the preferred strategy.

Among non-*BRCA* BC susceptibility genes, *PALB2*, *ATM*, and *CHEK2* showed the highest number of PVs. *PALB2* and *ATM* PVs were each identified in 1.3% of cases, consistent with previously reported prevalence rates (0.4–3.9% for *PALB2* [26,27], and in 1.1% for *ATM* [28]). *CHEK2* PVs were found in 1.9% of cases, or 1.4% when excluding lowPVs, aligning with the 1–2% reported in BC patients undergoing MGPT [28,29]. Single PVs were also identified in *BARD1* (0.1%), consistent with published prevalence values, ranging from 0.1% to 0.5% [4], and in *RAD51D* (0.1%), while *RAD51C* PVs were observed in 0.5% of cases. Notably, no PVs were found in the so-called syndromic genes, classified as high-penetrance BC genes, such as *TP53*, *PTEN*, or *STK11*.

Among other PFs identified through MGPT in cancer predisposition genes related to the patient’s phenotype, we included PVs in *MMR* genes detected in three patients with Lynch syndrome, biallelic PVs of *MUTYH* in a patient with attenuated adenomatous polyposis, and a PV in *CDKN2A* in a patient with a family history of melanoma. A clear association between these genes and BC risk has not yet emerged. Therefore, the BCs diagnosed in these patients may represent sporadic occurrences. However, particularly in the case of *MMR* genes, several studies have reported conflicting evidence, both supporting and refuting an association [30].

Finally, MGPT also led to the identification of SFs, such as two PVs in *CDH1* and one PV in *CDKN2A* in BC patients without a personal/family history suggestive of hereditary diffuse gastric cancer or melanoma and pancreatic carcinoma syndrome, respectively. In addition, a PV of *BRIP1* was found in two BC cases, with a frequency in line with the 0.3% previously reported [28]. *BRIP1* is currently known to be associated with an increased risk of OC [31]. To date, the evidence supporting a possible association with BC remains limited [28]. Accordingly, we decided to classify these results as SFs, considering that neither patient had a personal or family history of OC. Importantly, SFs pose significant challenges. Currently, specific guidelines for the management of SFs are lacking, and prospective studies are warranted to further define risk assessment and tailored clinical management strategies for carriers and their families. [32,33].

In our study, MGPT identified hetPVs in genes linked to autosomal recessive conditions, such as *MUTYH*, *NBN*, *RAD50*, and *MRE11A*—genes that have been proposed, but not confirmed as associated with hereditary BC risk [4,28,34]. In particular, we found a hetPV in *MUTYH* in 1.8% of patients. The c.1187G>A variant was the most common, occurring in 1% of patients, consistent with the frequency reported for the general population [35]. Although hetPVs currently bear limited clinical significance in terms of cancer risk assessment, their identification can aid in reproductive decision making. Accordingly, preconception counseling may be recommended for couples of reproductive age when a hetPV is identified through MGPT.

Furthermore, MGPT detected at least one lowPV in about 1.5% of cases, with the missense variant c.3920T>A (p.Ile1307Lys) in the *APC* gene identified in about 1% of cases, followed by three missense variants of *CHEK2*. The *APC* c.3920T>A variant doubles the risk of colorectal cancer in individuals of Ashkenazi Jewish descent [36], although its role in BC risk remains unclear [37]. Regarding *CHEK2*, loss-of-function PVs confer more than a two-fold BC risk [38]. This applies also for the c.1100del frameshift variant, one of the most frequent PVs encountered in our patients. On the other hand, the impact of missense variants is variable, since some of them are associated with lower risks (OR: 1.26–1.75) [4,25,39]. In particular, the c.470T>C (p.Ile157Thr), c.1283C>T (p.Ser428Phe), and c.1427C>T (p.Thr476Met) variants identified in our study are frequent alleles, with a very low association with BC [39,40]. Thus, lowPVs may generally contribute to BC risk, but genetic counseling should be carefully tailored. Indeed, intensive surveillance or surgery should be recommended only when a comprehensive personal/family assessment predicts a high BC risk.

As described, MGPT resulted in a significantly higher rate of VUSs compared to the rate for SGT. The identification of VUSs in MGPT presents a significant clinical challenge, as their pathogenicity remains uncertain due to limited or conflicting evidence. Their classification may evolve over time as new data accumulates through family segregation analyses, functional studies assessing the biological impact of the variants, in silico models, and the sharing of information via databases and registries [41,42]. Therefore, periodic reassessment is crucial for appropriate clinical management. The rate of VUSs in a given gene is influenced by several factors, including the penetrance of the associated disease, the availability of supporting information in the published literature, and, last but not least, the length of the gene coding sequence [43]. Interestingly, in our study, MGPT revealed the highest number of unique VUSs in *ATM*, followed by *BRCA2*, likely reflecting their large size.

Overall, MGPT, as well as whole exome and whole genome sequencing, generates considerably more data compared to SGT, posing challenges in interpreting and managing genetic testing results. With the rapid advancement of artificial intelligence, its application could represent a valuable tool for managing large testing volumes more efficiently and cost-effectively in the future. Nevertheless, the critical analysis and interpretation of genetic test results by clinicians and geneticists will remain indispensable to ensure accurate and responsible clinical decision making.

Notably, while SGT reduces the number of complex or uncertain results compared to those for MGPT, it may miss other actionable PVs in BC patients who receive uninformative results. Conversely, although MGPT extends testing to multiple genes, it does not entirely rule out the potential involvement of additional genes in patients with uninformative or uncertain results. In these cases, risk assessment should be based on the patient’s personal and family history.

Today, the dilemma of choosing between SGT and MGPT is nearing resolution, with growing support in favor of MGPT. However, to address the challenge of complex results, a reflex testing strategy could offer a promising middle ground. This approach would require close collaboration between medical and laboratory geneticists, along with an informed consent process that allows patients to indicate their preferences regarding results disclosure. Under this model, only PVs and VUSs in genes tied to the patient’s phenotype—as determined by the medical geneticist—would be reported, in line with the traditional SGT model. However, additional findings—such as SFs, hetPVs, or lowPVs in other genes included in the panel—could also be disclosed based on the patient’s preferences. This would ensure a more personalized and flexible approach to genetic testing. Importantly, the reflex model has not been analyzed in this study. Therefore, this promising proposal will require further studies to evaluate its efficiency and impact on the clinical management of genetic testing results for breast cancer patients.

Concerning clinicopathological features, multivariable analysis in patients undergoing SGT revealed that, as expected, TNBC was more prevalent in *BRCA* carriers, while ER-positive BC was less frequent. Similar associations were found for PVs in non-*BRCA* genes, even if after adjustment for multiple testing, they were no longer statistically significant. Of note, some non-*BRCA* genes are known to be more frequently associated with TNBC (e.g., *PALB2*, *BARD1*, *RAD51C*), and others are linked to ER-positive tumors (e.g., *ATM* and *CHEK2*) [4,25]. In line with the existing literature, we confirmed that ER-positive tumors were more common in *ATM*/*CHEK2* PV carriers.

Direct comparisons between *BRCA1* and *BRCA2* PV carriers revealed significant differences in molecular subtype and grading. Specifically, BCs in *BRCA1* PV carriers were more likely to be high-grade and triple-negative, whereas those in *BRCA2* PV carriers tended to be ER-positive, mainly luminal B HER2-negative, confirming previous findings in the literature [44,45].

From a molecular perspective, 6.5% of all PVs identified in the *BRCA* genes by SGT or MGPT were CNVs, consistent with published data [46]. Notably, *BRCA1* harbored the highest number of CNVs, probably due to the greater number of short interspersed nuclear elements repeats, particularly Alu repeats, which promote genomic rearrangements through non-allelic homologous recombination [47].

Regarding PVs in non-*BRCA* genes, MGPT identified CNVs only in the *PALB2* gene. Most of the *PALB2* PVs reported in the literature in BC patients are SNVs/indels. However, several CNVs have also been described, especially large deletions but also duplications [48,49,50,51]. In fact, similarly to the *BRCA1* gene, *PALB2* shows a high density of Alu elements [52]. Of relevance, the *PALB2* CNVs identified in this study were two duplications involving exons 11 and 13. Indeed, most CNVs reported in the literature span exons 7–13. Of note, this region encodes the WD40 domain, which is essential for the interaction with the BRCA2 protein [53]. Only recently, *PALB2* has been included in multigene panels used by clinical laboratories, and to date, only a few studies have systematically investigated the prevalence of *PALB2* CNVs in hereditary BCs or in the general population. In our study, *PALB2* CNVs accounted for 20% of the PVs identified in this gene, a higher frequency than the 10.3% reported in the literature [54].

Our study is not without its limitations, mainly due to its retrospective nature. Indeed, regarding the analyses of associations with clinicopathological features, we arbitrarily used different control groups for comparative analyses, including or excluding VUSs, hetPVs, and lowPVs from inconclusive results. This approach was chosen in order to increase the otherwise small size of the groups being compared, with the awareness that this may have introduced a bias. Indeed, our sample size does not allow for definitive conclusions. However, despite this limitation, our findings remain consistent with the previously reported literature. Notably, considering the small sample size and the multiple testing problem, we also presented results adjusted for false discovery rates. The sample size limitation could also be extended to the prevalence of CNVs in non-*BRCA* genes. Further studies with larger cohorts will be necessary to better define the contribution of CNVs in *PALB2* and other non-*BRCA* genes to hereditary BCs. Additionally, a selection bias may have influenced our overall results, as most patients underwent genetic testing due to a personal/family history suggestive of hereditary BC or other cancer predisposition syndromes. However, it is noteworthy that the selection criteria for these cases have evolved over time, becoming progressively more flexible. As a result, several patients were referred for genetic testing, even with a low probability of being PV carriers. Nevertheless, our results should not be considered representative of genetic testing outcomes in the general BC patient population.

## 5. Conclusions

Our results demonstrated that SGT can be considered the optimal initial strategy over MGPT for BC patients with a personal/family history strongly suggestive of *BRCA* gene involvement, significantly reducing the occurrence of uncertain and complex results compared to MGPT. Indeed, while MGPT detects a high rate of PVs, these do not necessarily constitute actionable findings. However, MGPT proves valuable in BC patients with a borderline phenotype, as evidenced by the high rate of primary findings in non-*BRCA* genes. In such cases, MGPT should be considered the most suitable test. Currently, the choice between SGT and MGPT is shifting in favor of MGPT. However, to address the management of its complex results, one potential strategy could involve a reflex model, in which complex or clinically less useful findings would be disclosed according to the patient’s preferences, as outlined in the informed consent. However, additional studies are required to evaluate the feasibility, efficiency, and impact of this model on clinical management.

Concerning clinicopathological features, our results confirmed associations previously reported in the literature, with *BRCA1* mainly associated with high-grade TNBCs, *BRCA2* with luminal B HER2-negative BCs, and *BRCA2*, *ATM*, and *CHEK2* with ER-positive BCs. Regarding PVs in non-*BRCA* genes, MGPT identified CNVs only in the *PALB2* gene. In our study, *PALB2* CNVs accounted for 20% of the PVs identified in this gene. Larger cohort studies are needed to further clarify the true contribution of CNVs in *PALB2* and other non-*BRCA* genes to hereditary breast cancers.

## Figures and Tables

**Table 1:** Genetic testing results obtained by SGT or MGPT approaches.

SGT Results		Patients (N = 308)	%
Positive		42	13.6%
	**PFs**	**42**	**13.6%**
	*BRCA1*	32	10.4%
	*BRCA2*	10	3.2%
Uncertain		21	6.8%
Uninformative		245	79.5%
**MGPT Results**		**Patients** **(N = 776)**	**%**
Positive		123	15.9%
	**PFs**	**89**	**11.5%**
	*BRCA1*	13	1.7%
	*BRCA2*	31	4.0%
	*BRCA1* + *BRCA2*	1	0.1%
	*BRCA2* + *BRCA2*	1	0.1%
	*BRCA2 + APC*	1	0.1%
	*BRCA2* + *CHEK2*	1	0.1%
	*BRCA2 + RAD51C*	1	0.1%
	*ATM*	9	1.2%
	*ATM* + *RAD51D*	1	0.1%
	*BARD1*	1	0.1%
	*CDH1*	1	0.1%
	*CDKN2A + MUTYH*	1	0.1%
	*CHEK2*	10	1.3%
	*MLH1*	2	0.3%
	*MUTYH* (biallelic)	1	0.1%
	*PALB2*	10	1.3%
	*PMS2*	1	0.1%
	*RAD51C*	3	0.4%
	**SFs**	**5**	**0.6%**
	*CDH1*	2	0.3%
	*CDKN2A*	1	0.1%
	*BRIP1*	2	0.3%
	**lowPVs**	**10**	**1.3%**
	*APC*	6	0.8%
	*CHEK2*	4	0.5%
	**hetPVs**	**18**	**2.3%**
	*MUTYH* (monoallelic)	13	1.7%
	*NBN + MRE11A*	1	0.1%
	*RAD50*	4	0.5%
	**lowPV + hetPV**	**1**	**0.1%**
	*APC + EPCAM*	1	0.1%
Uncertain		266	34.3%
Uninformative		387	49.9%

Positive results stratified by clinical significance are highlighted in bold. Abbreviations: hetPV, heterozygous PV in genes associated with autosomal recessive conditions; lowPV, low-penetrance PV; MGPT, multigene panel testing; PF, primary finding; PV, pathogenic/likely pathogenic variant; SF, secondary finding; SGT, single gene testing.

**Table 2:** Comparison between MGPT and SGT approaches in the 1084 patients analyzed.

Results	Overall (%)	SGT	MGPT	*p*-Value ^a^
Positive	165 (15.2%)	42 (13.6%)	123 (15.9%)	**<0.001**
Uncertain	287 (26.5%)	21 (6.8%)	266 (34.3%)	
Uninformative	632 (58.3%)	245 (79.5%)	387 (49.9%)	
PFs	131 (12.1%)	42 (13.6%)	89 (11.5%)	**<0.001**
Complex	321 (29.6%)	21 (6.8%)	300 (38.7%)	
Uninformative	632 (58.3%)	245 (79.5%)	387 (49.9%)	
	1084 (100%)	308 (100%)	776 (100%)	

^a^ The *p*-values in bold remained significant after adjustment for FDR (false discovery rate).

**Table 3:** Association between TNBC or ER status and genetic testing results from multivariate analysis in patients undergoing SGT or MGPT.

Variable	Contrast	Testing Approach	End-Point	OR	Low 95% CI	Up 95% CI	*p*-Value ^e^
Genetic testing results	Actionable vs. Inconclusive	SGT ^a^	TNBC ^b^	5.97	2.458	15.818	**<0.001**
			ER positive ^c^	0.29	0.115	0.677	**0.006**
		MGPT ^a^	TNBC ^b^	2.46	1.055	5.277	0.026
			ER positive ^d^	0.42	0.211	0.882	0.016

^a^ SGT actionable results included PVs in *BRCA* genes, while MGPT actionable results included PFs/SFs in non-*BRCA* genes. ^b^ Adjusted for bilateral tumor and grading. ^c^ Adjusted for grading. ^d^ Adjusted for bilateral tumor. CI, confidence interval; ER, estrogen receptor; OR, odds ratio; TNBC, triple-negative BC. ^e^ The *p*-values in bold remained significant after adjustment for FDR.

**Table 4:** ER or HER2 status in carriers and non-carriers of PVs in different BC genes.

	Overall Patients (N = 467)	*BRCA1/BRCA2* ^a^ Carriers (N = 47)	*ATM/CHEK2* ^b,c^ Carriers (N = 19)	*BARD1/* *PALB2/RAD51C* ^c^ Carriers (N = 14)	WT Patients (N = 387)	*p*-Value ^d^
**ER**						**0.004**
Positive	370 (79.2%)	35 (74.5%)	16 (84.2%)	6 (42.9%)	313 (80.9%)	
Negative	74 (15.8%)	10 (21.3%)	3 (15.8%)	7 (50.0%)	54 (14.0%)	
Unknown	23 (4.9%)	2 (4.3%)	0 (0.0%)	1 (7.1%)	20 (5.2%)	
**HER2**						0.599
Positive	52 (11.1%)	3 (6.4%)	3 (15.8%)	1 (7.1%)	45 (11.6%)	
Negative	374 (80.1%)	41 (87.2%)	14 (73.7%)	12 (85.7%)	307 (79.3%)	
Unknown	41 (8.8%)	3 (6.4%)	2 (10.5%)	1 (7.1%)	35 (9.0%)	

^a^ Only *BRCA* PV carriers identified by MGPT have been included; ^b^ *CHEK2* lowPVs have been excluded. ^c^ We excluded from this analysis three carriers of two PVs in different genes (namely, *ATM* and *RAD51D*, *BRCA2* and *CHEK2*, *BRCA2* and *RAD51C*). ^d^ The *p*-value in bold remained significant after adjustment for FDR. WT, wild-type.

## Data Availability

The data presented in this study are available upon request from the corresponding author. The data are not publicly available due to ethical restrictions.

## Supplementary Materials

The following supporting information can be downloaded at: https://www.mdpi.com/article/10.3390/cancers17091419/s1, Figure S1: Frequency of BC molecular subtypes in *BRCA1* (in blue) or *BRCA2* (in pink) carriers; Table S1: Genes included in the MGPT and their corresponding phenotype; Table S2: PVs detected by the MGPT approach; Table S3: Characteristics of the 1084 BC patients at the time of genetic testing; Table S4: Clinicopathological features of patients according to SGT results; Table S5: Clinicopathological features of patients according to MGPT results excluding *BRCA* carriers; Table S6: Unique PVs identified in the study population.

## Abbreviations

The following abbreviations are used in this manuscript:

ACMG	American College of Medical Genetics and Genomics
AMP	Association for Molecular Pathology
BC	breast cancer
*BRCA*	*BRCA1* and *BRCA2* genes
CI	confidence interval
CNV	copy number variant
CPS	cancer predisposition syndrome
ER	estrogen receptor
FDR	false discovery rate
hetPV	heterozygous PV in genes associated with autosomal recessive conditions
HGVS	Human Genome Variation Society
IARC	International Agency for Research on Cancer
IEO	European Institute of Oncology
indel	small insertion/deletion
IQR	interquartile range
lowPV	low-penetrance PV
LS	Lynch syndrome
MGPT	multigene panel testing
MMR	mismatch repair
NGS	next generation sequencing
NST	no special type
OC	ovarian cancer
OR	odds ratio
PF	primary finding
PV	pathogenic/likely pathogenic variant
SF	secondary finding
SGT	single gene testing
SNV	single nucleotide variant
TNBC	triple-negative breast cancer
VUS	variant of uncertain significance
WT	wild-type
