# Supplementary material for: Germline Testing in Breast Cancer: A Single-Center Analysis Comparing Strengths and Challenges of Different Approaches
Source: Cancers (Basel). 2025 Apr 24;17(9):1419. doi: 10.3390/cancers17091419 (PMC12071043; doi:10.3390/cancers17091419)
Supplement: Supplementary file 1 [file cancers-17-01419-s001.zip › Table S2.pdf]

Table S2. PVs detected by the MGPT approach.

Genes	N. PV/patient	N. patients	Total N. PVs
APC	1	6	6
ATM	1	9	9
BARD1	1	1	1
BRCA1	1	13	13
BRCA2	1	31	31
BRIP1	1	2	2
CDH1	1	3	3
CDKN2A	1	1	1
CHEK2	1	14	14
MLH1	1	2	2
MUTYH	1	13	13
PALB2	1	10	10
PMS2	1	1	1
RAD50	1	4	4
RAD51C	1	3	3
BRCA1+BRCA2	2	1	2
BRCA2+BRCA2	2	1	2
BRCA2+CHEK2	2	1	2
BRCA2+RAD51C	2	1	2
BRCA2+APC	2	1	2
ATM+RAD51D	2	1	2
MUTYH+MUTYH	2	1	2
NBN+MRE11A	2	1	2
CDKN2A+MUTYH	2	1	2
APC+EPCAM	2	1	2
		123	133

MGPT, multigene panel testing; PV, pathogenic/likely pathogenic variant.
