# Supplementary material for: Germline Testing in Breast Cancer: A Single-Center Analysis Comparing Strengths and Challenges of Different Approaches
Source: Cancers (Basel). 2025 Apr 24;17(9):1419. doi: 10.3390/cancers17091419 (PMC12071043; doi:10.3390/cancers17091419)
Supplement: Supplementary file 1 [file cancers-17-01419-s001.zip › Table S3.pdf]

Table S3. Characteristics of the 1084 BC patients at the time of genetic testing.

	Overall (N=1084)	SGT (N=308)	MGPT (N=776)	*p*-Value [d]
**Gender**				
Female	1068 (98.5%)	297 (96.4%)	771 (99.4%)	**<0.001**
Male	16 (1.5%)	11 (3.6%)	5 (0.6%)	
**Age at diagnosis** [a]				
Median (IQR)	45 (39, 51)	46 (38, 53)	45 (39, 51)	0.353
**Bilateral BC**				
Yes	151 (13.9%)	34 (11.0%)	117 (15.1%)	0.083
No	933 (86.1%)	274 (89.0%)	659 (84.9%)	
**Histotype** [a]				
NST	811 (74.8%)	260 (84.4%)	551 (71.0%)	**<0.001**
ILC	127 (11.7%)	16 (5.2%)	111 (14.3%)	
Other	130 (12.0%)	31 (10.1%)	99 (12.8%)	
Unknown	16 (1.5%)	1 (0.3%)	15 (1.9%)	
**Grading** [a]				
1-2	593 (54.7%)	154 (50.0%)	439 (56.6%)	**0.003**
3	371 (34.2%)	130 (42.2%)	241 (31.1%)	
Unknown	120 (11.1%)	24 (7.8%)	96 (12.4%)	
**TNBC**				
Yes	175 (16.1%)	88 (28.6%)	87 (11.2%)	**<0.001**
No	867 (80.0%)	212 (68.9%)	655 (84.4%)	
Unknown	42 (3.9%)	8 (2.6%)	34 (4.4%)	
**ER** [a]				
Positive	820 (75.6%)	199 (64.6%)	621 (80.0%)	**<0.001**
Negative	223 (20.6%)	102 (33.1%)	121 (15.6%)	
Unknown	41 (3.8%)	7 (2.3%)	34 (4.4%)	
**Molecular subtype** [a]				
Luminal type	794 (73.2%)	193 (62.7%)	601 (77.4%)	**<0.001**
HER2-enriched [b]	37 (3.4%)	12 (3.9%)	25 (3.2%)	
TNBC	175 (16.1%)	88 (28.6%)	87 (11.2%)	
Unknown	78 (7.2%)	15 (4.9%)	63 (8.1%)	
**Other *BRCA*-related tumors** [c]				
Yes	18 (1.7%)	8 (2.6%)	10 (1.3%)	0.208
No	1066 (98.3%)	300 (97.4%)	766 (98.7%)	
**Positive FH of BC**				
Yes	694 (64.0%)	181 (58.8%)	513 (66.1%)	**<0.001**
No	340 (31.4%)	126 (40.9%)	214 (27.6%)	
Unknown	50 (4.6%)	1 (0.3%)	49 (6.3%)	
**Positive FH of OC**				
Yes	102 (9.4%)	45 (14.6%)	57 (7.3%)	**0.001**
No	932 (86.0%)	262 (85.1%)	670 (86.3%)	
Unknown	50 (4.6%)	1 (0.3%)	49 (6.3%)	
**≥ 1 family member with bilateral BC**				
Yes	67 (6.2%)	13 (4.2%)	54 (7.0%)	0.077
No	967 (89.2%)	294 (95.5%)	673 (86.7%)	
Unknown	50 (4.6%)	1 (0.3%)	49 (6.3%)	
**≥ 1 family member with male BC**				

Yes	11 (1.0%)	3 (1.0%)	8 (1.0%)	
No	1023 (94.4%)	304 (98.7%)	719 (92.7%)	1
Unknown	50 (4.6%)	1 (0.3%)	49 (6.3%)	
**≥ 1 family member with BC and OC**				
Yes	12 (1.1%)	6 (1.9%)	6 (0.8%)	
No	1022 (94.3%)	301 (97.7%)	721 (92.9%)	0.199
Unknown	50 (4.6%)	1 (0.3%)	49 (6.3%)	

[a] referred to the first (or only) BC. In case of bilateral synchronous tumor, in this description priority was given to invasive tumors and triple-negative tumors. [b] HER2-enriched is defined as hormone receptor-negative and HER2 positive tumor. [c] Other *BRCA*-related tumors include ovarian, prostate, and pancreatic cancers. [d] The p-Values in bold remained significant after adjustment for FDR. BC, breast cancer; FDR, false discovery rate; FH, family history; ILC, invasive lobular carcinoma; NST, no special type; OC, ovarian cancer; TNBC, triple-negative breast cancer.
