# Supplementary material for: Germline Testing in Breast Cancer: A Single-Center Analysis Comparing Strengths and Challenges of Different Approaches
Source: Cancers (Basel). 2025 Apr 24;17(9):1419. doi: 10.3390/cancers17091419 (PMC12071043; doi:10.3390/cancers17091419)
Supplement: Supplementary file 1 [file cancers-17-01419-s001.zip › Table S4.pdf]

**Table S4.** Clinicopathological features of patients according to SGT results.

| | Overall patients (N=308) | Actionable result [a] (N=42) | Inconclusive result [a] (N=266) | *p*-Value [d] |
|---|---|---|---|---|
| **Gender** | | | | |
| Female | 297 (96.4%) | 42 (100%) | 255 (95.9%) | 0.372 |
| Male | 11 (3.6%) | 0 (0.0%) | 11 (4.1%) | |
| **Age at diagnosis** [b] | | | | |
| Median (IQR) | 46 (38-53) | 45 (38-53) | 46 (38-53) | 0.580 |
| **Bilateral BC** | | | | |
| Yes | 34 (11.0%) | 6 (14.3%) | 28 (10.5%) | 0.434 |
| No | 274 (89.0%) | 36 (85.7%) | 238 (89.5%) | |
| **Histotype** [b] | | | | |
| NST | 260 (84.4%) | 39 (92.9%) | 221 (83.1%) | |
| ILC | 16 (5.2%) | 2 (4.8%) | 14 (5.3%) | 0.189 |
| Other | 31 (10.1%) | 1 (2.4%) | 30 (11.3%) | |
| Unknown | 1 (0.3%) | 0 (0.0%) | 1 (0.4%) | |
| **Grading** [b] | | | | |
| 1-2 | 154 (50.0%) | 7 (16.7%) | 147 (55.3%) | **<0.001** |
| 3 | 130 (42.2%) | 31 (73.8%) | 99 (37.2%) | |
| Unknown | 24 (7.8%) | 4 (9.5%) | 20 (7.5%) | |
| **TNBC** | | | | |
| Yes | 88 (28.6%) | 30 (71.4%) | 58 (21.8%) | **<0.001** |
| No | 212 (68.9%) | 12 (28.6%) | 200 (75.2%) | |
| Unknown | 8 (2.6%) | 0 (0.0%) | 8 (3.0%) | |
| **ER** [b] | | | | |
| Positive | 199 (64.6%) | 12 (28.6%) | 187 (70.3%) | **<0.001** |
| Negative | 102 (33.1%) | 30 (71.4%) | 72 (27.1%) | |
| Unknown | 7 (2.3%) | 0 (0.0%) | 7 (2.6%) | |
| **HER2** [b] | | | | |
| Positive | 38 (12.3%) | 1 (2.4%) | 37 (13.9%) | 0.049 |
| Negative | 254 (82.5%) | 41 (97.6%) | 213 (80.1%) | |
| Unknown | 16 (5.2%) | 0 (0.0%) | 16 (6.0%) | |
| **Molecular subtype** [b] | | | | |
| Luminal | 193 (62.7%) | 12 (28.6%) | 181 (68.0%) | |
| HER2+ | 12 (3.9%) | 0 (0.0%) | 12 (4.5%) | **<0.001** |
| TNBC | 88 (28.6%) | 30 (71.4%) | 58 (21.8%) | |
| Unknown | 15 (4.9%) | 0 (0.0%) | 15 (5.6%) | |
| **Other *BRCA*-related tumor** [c] | | | | 0.299 |
| Yes | 8 (2.6%) | 2 (4.8%) | 6 (2.3%) | |
| No | 300 (97.4%) | 40 (95.2%) | 260 (97.7%) | |
| **Positive FH of BC** | | | | |
| Yes | 181 (58.8%) | 25 (59.5%) | 156 (58.6%) | >0.999 |
| No | 126 (40.9%) | 17 (40.5%) | 109 (41.0%) | |
| Unknown | 1 (0.3%) | 0 (0.0%) | 1 (0.4%) | |
| **Positive FH of OC** | | | | |
| Yes | 45 (14.6%) | 13 (31.0%) | 32 (12.0%) | **0.003** |
| No | 262 (85.0%) | 29 (69.0%) | 233 (87.6%) | |
| Unknown | 1 (0.3%) | 0 (0.0%) | 1 (0.4%) | |

| | | | | |
|---|---|---|---|---|
| **≥ 1 family member with bilateral BC** | | | | |
| Yes | 13 (4.2%) | 2 (4.8%) | 11 (4.1%) | |
| No | 294 (95.5%) | 40 (95.2%) | 254 (95.5%) | 0.694 |
| Unknown | 1 (0.3%) | 0 (0.0%) | 1 (0.4%) | |
| **≥ 1 family member with male BC** | | | | |
| Yes | 3 (1.0%) | 0 (0.0%) | 3 (1.1%) | |
| No | 304 (98.7%) | 42 (100%) | 262 (98.5%) | >0.999 |
| Unknown | 1 (0.3%) | 0 (0.0%) | 1 (0.4%) | |
| **≥ 1 family member with BC and OC** | | | | |
| Yes | 6 (1.9%) | 3 (7.1%) | 3 (1.1%) | |
| No | 301 (97.7%) | 39 (92.9%) | 262 (98.5%) | 0.035 |
| Unknown | 1 (0.3%) | 0 (0.0%) | 1 (0.4%) | |

[a] actionable and inconclusive results refer to PV carriers and non-carriers in *BRCA* genes, respectively, [b] referred to the first (or only) breast tumor. In case of bilateral synchronous tumor, in this description priority was given to infiltrating tumors and triple-negative tumors. [c] Other *BRCA*-related tumors include ovarian, prostate, and pancreatic cancers. [d] The *p*-Values in bold remained significant after adjustment for FDR. ER, estrogen receptor; FDR, false discovery rate; ILC, invasive lobular carcinoma; IQR, interquartile range; OC, ovarian cancer; SGT, single gene testing.
