# Supplementary material for: Germline Testing in Breast Cancer: A Single-Center Analysis Comparing Strengths and Challenges of Different Approaches
Source: Cancers (Basel). 2025 Apr 24;17(9):1419. doi: 10.3390/cancers17091419 (PMC12071043; doi:10.3390/cancers17091419)
Supplement: Supplementary file 1 [file cancers-17-01419-s001.zip › Table S5.pdf]

Table S5. Clinicopathological features of patients according to MGPT results excluding *BRCA* carriers.

	Overall patients (N=727)	Actionable result [a] (N=45)	Inconclusive result [a] (N=682)	*p*-Value [d]
**Gender**				
Female	722 (99.3%)	45 (100%)	677 (99.3%)	>0.999
Male	5 (0.7%)	0 (0.0%)	5 (0.7%)	
**Age at diagnosis** [b]				
Median (IQR)	45 (42-52)	45 (39-51)	45 (39-51)	0.300
**Bilateral BC**				
Yes	105 (14.4%)	9 (20.0%)	96 (14.1%)	0.381
No	622 (85.6%)	36 (80.0%)	586 (85.9%)	
**Histotype** [b]				
NST	512 (70.4%)	33 (73.3%)	479 (70.2%)	
ILC	107 (14.7%)	5 (11.1%)	102 (15.0%)	0.850
Other	96 (13.2%)	5 (11.1%)	91 (13.3%)	
Unknown	12 (1.7%)	2 (4.4%)	10 (1.5%)	
**Grading** [b]				
1-2	420 (57.8%)	18 (40.0%)	402 (58.9%)	**0.011**
3	216 (29.7%)	21 (46.7%)	195 (28.6%)	
Unknown	91 (12.5%)	6 (13.3%)	85 (12.5%)	
**TNBC**				
Yes	77 (10.6%)	9 (20.0%)	68 (10.0%)	0.039
No	619 (85.1%)	33 (73.3%)	586 (85.9%)	
Unknown	31 (4.3%)	3 (6.7%)	28 (4.1%)	
**ER** [b]				
Positive	586 (80.6%)	30 (66.7%)	556 (81.5%)	0.036
Negative	111 (15.3%)	12 (26.7%)	99 (14.5%)	
Unknown	30 (4.1%)	3 (6.7%)	27 (4.0%)	
**HER2** [b]				
Positive	90 (12.4%)	7 (15.6%)	83 (12.2%)	0.466
Negative	576 (79.2%)	32 (71.1%)	544 (79.8%)	
Unknown	61 (8.4%)	6 (13.3%)	55 (8.1%)	
**Molecular subtype** [b]				
Luminal	567 (78.0%)	30 (66.7%)	537 (78.7%)	
HER2+	25 (3.4%)	2 (4.4%)	23 (3.4%)	0.075
TNBC	77 (10.6%)	9 (20.0%)	68 (10.0%)	
Unknown	58 (8.0%)	4 (8.9%)	54 (7.9%)	
**Other tumor** [c]				
Yes	57 (7.8%)	7 (15.6%)	50 (7.3%)	0.076
No	670 (92.2%)	38 (84.4%)	632 (92.7%)	
**Positive FH of BC**				
Yes	474 (65.2%)	32 (71.1%)	442 (64.8%)	0.989
No	204 (28.1%)	13 (28.9%)	191 (28.0%)	
Unknown	49 (6.7%)	0 (0.0%)	49 (7.2%)	
**Positive FH of OC**				
Yes	47 (6.5%)	3 (6.7%)	44 (6.5%)	>0.999
No	631 (86.8%)	42 (93.3%)	589 (86.4%)	
Unknown	49 (6.7%)	0 (0.0%)	49 (7.2%)	
**≥ 1 family member with bilateral BC**				

Yes	52 (7.2%)	6 (13.3%)	46 (6.7%)	
No	626 (86.1%)	39 (86.7%)	587 (86.1%)	0.145
Unknown	49 (6.7%)	0 (0.0%)	49 (7.2%)	
**≥ 1 family member with male BC**				
Yes	5 (0.7%)	1 (2.2%)	4 (0.6%)	
No	673 (92.6%)	44 (97.8%)	629 (92.2%)	0.291
Unknown	49 (6.7%)	0 (0.0%)	49 (7.2%)	
**≥ 1 family member with BC and OC**				
Yes	2 (0.3%)	0 (0.0%)	2 (0.3%)	
No	676 (93.0%)	45 (100%)	631 (92.5%)	>0.999
Unknown	49 (6.7%)	0 (0.0%)	49 (7.2%)	

[a] actionable and inconclusive results refer to patients with or without PFs/SFs, excluding PV *BRCA* carriers.

[b] referred to the first (or only) breast tumor, [c] cancers related to predisposition genes included in the multigene panel, *i.e.*, cancers of the colorectum, ovary, pancreas, kidney, stomach, thyroid, uterus, bladder, sarcomas, and melanomas. [d] The *p*-Values in bold remained significant after adjustment for FDR.
