# Supplementary figures and images for: Germline Testing in Breast Cancer: A Single-Center Analysis Comparing Strengths and Challenges of Different Approaches
Source: Cancers (Basel). 2025 Apr 24;17(9):1419. doi: 10.3390/cancers17091419 (PMC12071043; doi:10.3390/cancers17091419)

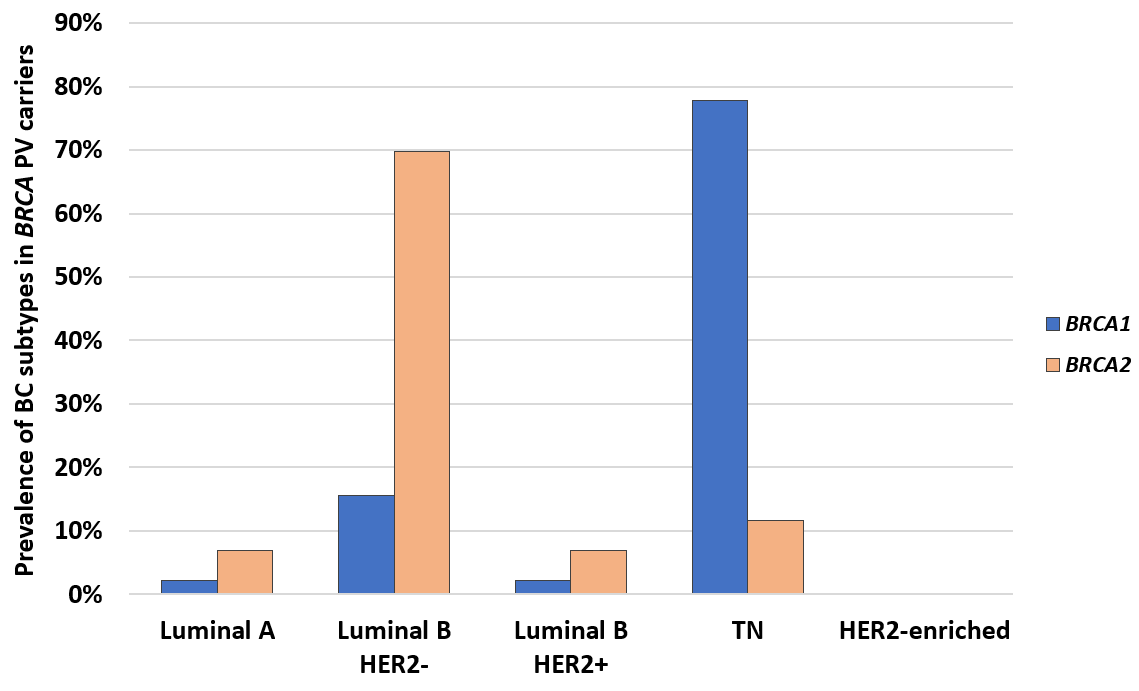

Supplement: Supplementary file 1 [file cancers-17-01419-s001.zip › Figure S1.PNG]
